# Supplementary material for: Enhancing Brain Age Prediction and Neurodegeneration Detection with Contrastive Learning on Regional Biomechanical Properties
Source: bioRxiv. 2025 Mar 26:2025.03.25.645330. Preprint. [Version 1] doi: 10.1101/2025.03.25.645330 (PMC11974862; doi:10.1101/2025.03.25.645330)
Supplement: Supplement 1 [file NIHPP2025.03.25.645330v1-supplement-1.pdf]

bioRxiv preprint doi: https://doi.org/10.1101/2025.03.25.645330; this version posted March 26, 2025. The copyright holder for this preprint (which was not certified by peer review) is the author/funder, who has granted bioRxiv a license to display the preprint in perpetuity. It is made available under aCC-BY 4.0 International license.

Supplementary Materials & Methods

Localized Aging Effects in Spatially Normalised Mechanical Properties Confirm MRE's Sensitivity Beyond Global Trends

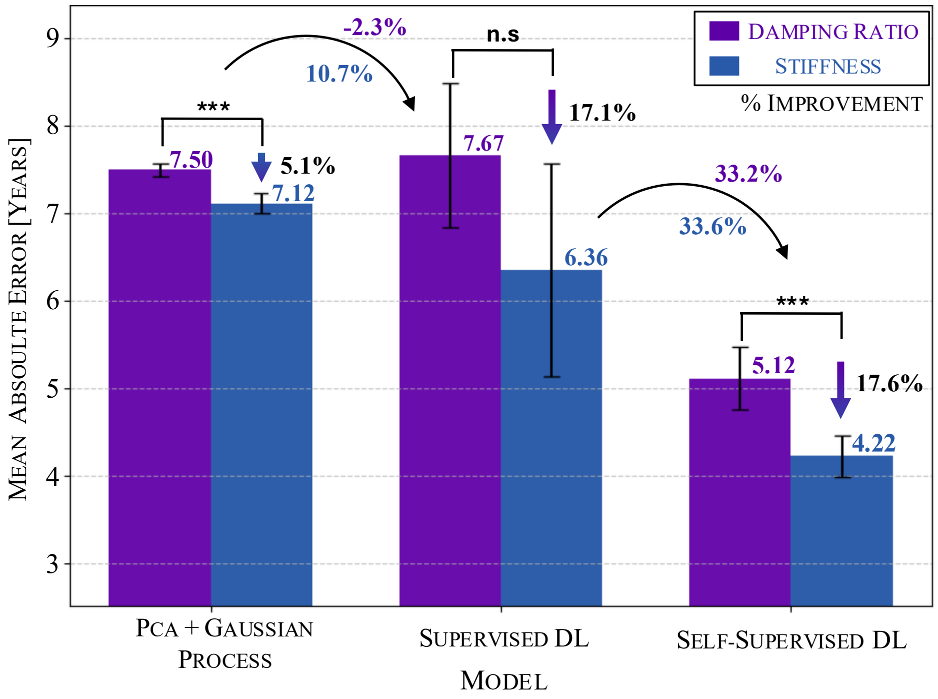

Supplementary Figure 1: Disentangling Global and Local Aging Effects: Impact of Spatial Normalisation on MRE-Based Predictions. *Evaluating spatially normalised mechanical properties highlights the predictive value of localized aging effect. Stiffness proves to be the stronger predictor in all spatially normalised models.*

To better understand the effects of global versus local aging patterns, we evaluated brain age prediction using spatially normalised MRE scans (see Supplementary Fig. S1). Unlike our primary analysis, which retained global trends in stiffness and damping ratio, this approach normalises each scan to zero mean and unit variance before training. By removing large-scale mechanical aging trends, this normalisation isolates spatially localised aging effects. Under the spatially normalised conditions, stiffness outperforms damping ratio for kernel-based methods. PCA+GPs achieve an MAE of 7.12 years for stiffness, a 5.1% improvement over damping ratio (MAE = 7.50 years). Similarly, in deep learning models, stiffness emerges as the stronger predictor. Supervised learning reduces the MAE to 6.36 years for stiffness compared to 7.67 years for damping ratio, while self-supervised learning further improves stiffness-based predictions to an MAE of 4.22 years. Compared to the previous normalisation method, which preserved global aging trends, supervised and self-supervised deep learning models show slightly higher MAEs, highlighting the

bioRxiv preprint doi: https://doi.org/10.1101/2025.03.25.645330; this version posted March 26, 2025. The copyright holder for this preprint (which was not certified by peer review) is the author/funder, who has granted bioRxiv a license to display the preprint in perpetuity. It is made available under aCC-BY 4.0 International license.

predictive value of large-scale mechanical changes. However, this analysis shows that spatially localized distribution changes contain key information for brain age prediction.

Pooled Dataset Exhibits Bi-Modality Age Distribution

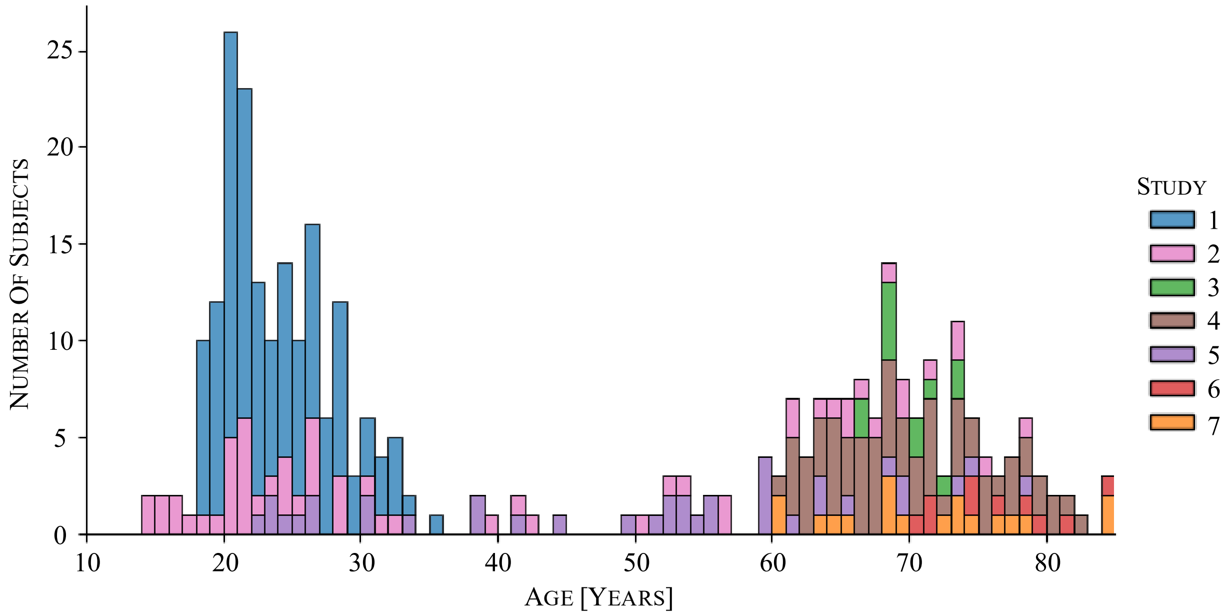

Supplementary Figure 2: Age Distribution of Pooled Studies of MRE Dataset. *Contribution of each study is highlighted in colour. The distribution shows bi-modal characteristics with two predominant age groups among samples.*

Understanding the age distribution of our dataset is essential for evaluating the generalizability of brain age prediction models. Supplementary Fig. S2 presents the combined age distribution from all pooled studies contributing to the MRE dataset, with each study's contribution highlighted in different colours. The distribution exhibits a bi-modal pattern, characterised by two predominant age clusters corresponding to younger and older cohorts. This pattern arises due to the inherent limitation of a lack of middle-aged volunteers in clinical trials. To mitigate this imbalance, we employ the adaptive neighbourhood approach, which compensates for dataset non-uniformity through the contrastive regression loss function. This method ensures that age predictions remain robust despite the dataset’s skewed age distribution, enhancing the model's ability to generalize across age groups.
